# Understanding the genetic complexity of *Leishmania infantum* in the Americas: a focus on 3’NT/NU gene deletion

**DOI:** 10.1590/0074-02760240160

**Published:** 2025-03-24

**Authors:** Monique Florêncio, Elisa Cupolillo, Mariana Côrtes Boité

**Affiliations:** 1 Fundação Oswaldo Cruz-Fiocruz, Instituto Oswaldo Cruz, Laboratório de Pesquisa em Leishmanioses, Rio de Janeiro, RJ, Brasil

**Keywords:** American visceral Leishmaniasis, Leishmania infantum, deletion, ecto-3’-nucleotidase/nuclease, adaptation, evolutionary process

## Abstract

Visceral Leishmaniasis in the Americas is primarily associated with *Leishmania* (*Leishmania*) *infantum*. This parasite is non-native and was imported during the colonisation era. The constitutive instability of the *Leishmania* genome allows this parasite to express flexibility in adapting to environmental fluctuations and different selective pressures, such as those the parasite faced when arrived in the New World. Therefore, genetic diversity is expected among the populations of *L. infantum* in the Americas, despite the bottle neck of importation route. Indeed, subpopulation of strains of *L. infantum* carrying a homozygous deletion in the genome was detected exclusively in the continent. These strains are more spread across Brazilian territory to the detriment of the non-deleted; the locus includes four genes, two of which encode the enzyme ecto-3’-nucleotidase/nuclease (3’NT/NU), a virulence factor in *L. infantum*. In this review, we highlight the sub estimated genetic complexity of *L. infantum* populations in Brazil, addressing the biological importance of the 3’NT/NU enzyme and the possible phenotypic impacts of its deletion, pointing out how it may configure an adaptive strategy for *L. infantum*. Finally, we raise the discussion of how the genome of *L. infantum* might be shaped in a unique way under the ecological conditions of Brazil.

Leishmaniasis has three main clinical forms: visceral, cutaneous and mucocutaneous. According to the World Health Organization (WHO), constitutes a major public health problem around the globe, mainly affecting poverty-stricken regions. It is endemic in 92 countries, of which 72 are developing countries, and where one billion people are living at risk of contracting the disease.^1^ In the New World (NW), Brazil leads the number of cases.^2^ ^,^ ^3^ Leishmaniasis is an ancient disease, with several primitive descriptions found in archaeological findings and in molecular analyses of mummified human remains.^4^ ^,^ ^5^ ^,^ ^6^ There are three hypotheses to explain the origin of the genus *Leishmania*, all under ongoing debate.^5^ One of the most accepted is the supercontinent hypothesis, proposing that during the separation of Gondwana in the Mesozoic era, the subgenus *Viannia* evolved in South America, while the subgenera *Sauroleishmania* and *Leishmania* developed in Africa.^7^ Enzymatic and molecular analyses have corroborated that the origin of the agent of visceral leishmaniasis in the Americas - *L. infantum*, occurred during the period of European colonisation, approximately 500 years ago.^7^ ^,^ ^8^ ^,^ ^9^ It is a parasite imported by the colonisers. It is highly probably that there were multiple introductions of these trypanosomatids carried by asymptomatic dogs and other mammalian hosts. Once in the NW, this parasite found appropriate ecological conditions for transmission, such as the presence of its main urban reservoir, *Canis familiaris*, but with important differences from those found in the Old World (OW), such as the existence of permissive sandfly vectors. The scenario favoured parasite adaptation and the establishment of new transmission cycles.^10^ Therefore, these small founding populations of *L. infantum* have been facing selective pressures, distinct from those found in the OW, as well as unknown sampling effects that drive genetic drift.^11^ In addition to this historical epidemiological scenario and the evolutionary processes that permeate the parasite arrival in the NW, is the fact that *Leishmania* presents constitutive genomic adaptability. Among the molecular traits involved in such characteristics are: (i) the mosaic aneuploidy, a phenomenon where the number of copies of individual chromosomes varies within and between cells of a clonal population,^12^ ^,^ ^13^ configuring itself not only as a product of genomic plasticity, but as a reflection of this inherited trait, and (ii) a complex and predominantly clonal life cycle, presenting hybridisation events in an unknown frequency^14^ ^,^ ^15^ and a close ecological relationship with its hosts.^16^ ^,^ ^17^ ^,^ ^18^ Such characteristics are of great relevance for the biology and adaptability of the parasite and thus for the associated eco-epidemiological scenario. For instance, intra-specific genetic variation can be associated with the main differences in disease pathology^19^ and drug resistance.^18^ Importantly, losing genes is also a significant adaptive strategy for *Leishmania*.^13^ In this regard, Carnielli et al.,^20^ through a genome-wide association study (GWAS), identified populations of *L. infantum* in Minas Gerais, Maranhão and Piauí (Brazil) carrying a large deletion (> 12 kb) found in the often reported as a one of the most stable chromosome within *Leishmania* mosaic aneuploidy, the polysomic chromosome 31 (chr31)^18^ ^,^ ^20^ ^,^ ^21^ (as depicted in Figure). The genomic trait was associated with the phenotype of resistance to an important leishmanicidal drug, miltefosine, and thus named miltefosine sensitivity locus (MSL).^20^ The homozygous deletion covers four open reading frames: LinJ.31.2370 (ecto-3′-nucleotidase/nuclease), LinJ.31.2380 (ecto-3′-nucleotidase/nuclease precursor), LinJ.31.2390 (helicase-like protein), and LinJ.31.2400 (3,2-trans-enoyl-CoA isomerase). Afterwards, Schwabl et al.^11^ performed a comparative analysis of a wide panel of *L. infantum* genomes [107 from the NW and 19 from the OW, plus 75 additional samples from the NW by quantitative polymerase chain reaction (qPCR)], confirming the occurrence of the deletion. The deletion-carrying strains (Del) were found exclusively among American samples (mainly from Brazil - 126 of 177), while all available OW genomes examined were non-deleted (NonDel).^11^ Remarkably, Del parasites were more frequent and geographically widespread then NonDel strains. To this peculiar finding adds the functional description of two deleted copies within the site, encoding the enzyme ecto-3’-nucleotidase/nuclease (3’NT/NU). The enzyme plays an important role in parasite nutrition, establishment of infection in vertebrate host,^22^ uptake of purines,^23^ escape of parasites from neutrophil networks (NETs)^24^ and the ability to infect macrophages.^25^ ^,^ ^26^ Therefore, this enzyme is expected to be quite relevant for the parasite in the initial stages of infection and constitutes a virulence factor for *L. infantum*.^22^ ^,^ ^23^ Despite its projected importance, it has been confirmed that Del strains indeed lack 3’Nu activity *in vitro*.^11^ An open question, therefore, is how these deficient parasites are dispersing more successfully across Brazilian territory to the detriment of the NonDel strains, and what are, if any, the associated epidemiological consequences. One hypothesis is that the deletion unexpectedly increases parasite fitness in Brazilian ecological landscape. In this review, we discuss the aspects that may relate to the spread of deletion-carrying strains, possible implications of the deleted locus on the virulence, infectivity, and transmissibility of *L. infantum* in Brazil, and the epidemiological consequences of this scenario.

**Figure:**
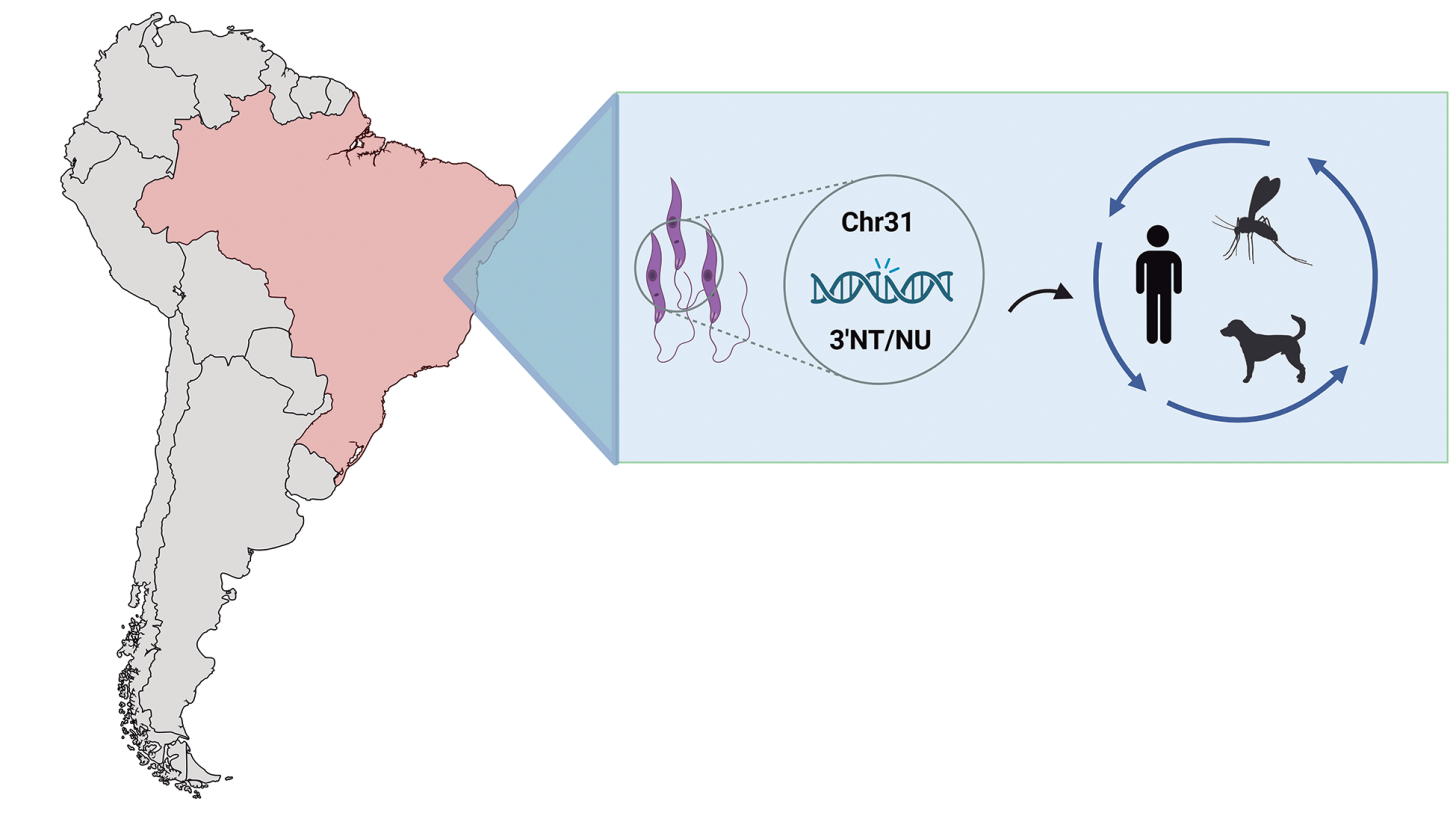
A subpopulation of *Leishmania infantum* strains with a homozygous deletion in the genome was detected exclusively in the Americas, predominantly in Brazil, at the expense of non-deleted strains. Among the deleted genes, two encode the enzyme ecto-3’-nucleotidase/nuclease (3’NT/NU), a known virulence factor in *L. infantum*. The epidemiological consequences and implications for the transmission cycle remain an open question, and represent a unique opportunity to advance our understanding of adaptive and evolutionary molecular strategies in *Leishmania*.

**Aspects on the ecological scenario of *L. infantum* population in Brazil**

Upon arriving in the Americas, *L. infantum* had to adapt to different, but suitable ecological conditions to establish new transmission cycles. This includes NW vertebrate hosts such as dogs in the domestic and peri-urban environment and sylvatic animals: endodentates, carnivores, marsupials, rodents, and primates,^27^ mostly diverse from the wildlife species that have been parasitised in the OW.^28^ The main vector species in the Americas is *Lutzomyia longipalpis*, a sandfly belonging to a different genus from that of the OW, *Phlebotomus*.^29^ The climatic conditions in NW are also distinct, favouring sandflies development trough out the year,^28^ unlike the scenario observed in the Mediterranean region, where the vector population presents a more defined seasonal pattern, typically from spring to autumn.^30^ Moreover, *Lu. longipalpis* exhibits a complex population structure, evidenced by chemical, behavioural and molecular traits.^31^ More recently, in Brazil, other sandfly species have been pointed as vectors, such as *Lu. cruzi* ^32^ or as putative vector, such as *Lu. migonei.* ^33^ This fact, combined to the American biomes, distinct from those in Europe, have likely contributed to the establishment of locally specific transmission cycles of the American visceral leishmaniasis (AVL).^34^ Several studies have shown that it is probable that the transmission, virulence, and clinical outcome are strongly influenced by coevolutionary interactions between the parasite species and specific sandfly genotypes.^35^ ^,^ ^36^ Thus, constitutive genomic characteristics of *L. infantum* (syn. *Leishmania chagasi*) might emerge as a reflex of the environmental selective pressures to which they are subjected to. In Brazil, Ferreira et al.^37^ examined by multilocus microsatellite typing (MLMT) 162 samples of *L. infantum* from dogs and humans, and a sample of fox (*Cerdocyon* sp.) and opossum (*Didelphis marsupialis*), from most Brazilian states and Paraguay. Despite the homogeneity expressed by multilocus enzyme electrophoresis (MLEE), MLMT exposed the circulation of three distinct parasite populations: POP1 composed of 111 strains and 36 genotypes, POP2 with 31 strains and 19 genotypes and POP3 with 31 strains and 12 genotypes. Curiously, the geographical distribution of these strains was not homogeneous. POP1 was found in 16 Brazilian states and in Paraguay, being predominant in some geographic areas, such as in Espírito Santo, where 95% of the strains were assigned POP1. In contrast, POP2 was found in 10 Brazilian states, but predominant only in Mato Grosso. POP3 was predominant in Mato Grosso do Sul (75%). When overlapped with reports of sandflies, one finds that *Lu. cruzi*, a closely related species to *Lu. longipalpis*,^38^ ^,^ ^39^ participates in transmission cycle of *L. infantum* in Mato Grosso and Mato Grosso do Sul.^39^ ^,^ ^40^ It is plausible, thus, to assume there is a parasite-sandfly association in that area. Schwabl et al.^11^ found data suggesting a similar pattern after screening the occurrence of Del *L. infantum* strains. Authors found the non-deleted strains (NonDel) were highly concentrated and frequent in the state of Mato Grosso do Sul, which, again, may be related to the alternative vector in this region, *Lu. cruzi*,^11^ or to the alternative introduction of parasites to that region by the bordering countries (*e.g.*, Bolivia). Genetic exchange was already demonstrated for many *Leishmania* parasites, a process occurring manly, if not exclusively, in the sandflies.^14^ ^,^ ^15^ ^,^ ^41^ Contradicting the idea that the *L. infantum* population in Brazil is homogeneous and increasing the complexity of the population structure of this parasite, six heterozygous samples (HTZ) were described and confirmed as hybrids originated from Del and NonDel in contact zone regions.^11^ Therefore, and considering that parasite itself play a key role in clinical outcome,^10^ ^,^ ^11^ it is plausible to assume the epidemiological variations current described in AVL are not restrictedly linked to host and environment traits. The parasite, thus, is a major player to be considered, and the relatively short time *L. infantum* inhabits the continent should not be underestimated during epidemiological surveillance approaches.

**Adaptation strategies and fitness gain in *Leishmania* vs the dispersion scenario of Del parasites**

Schwabl et al.^11^ pointed out that the sub-chromosomal deletion found among American strains was possibly an inherited trait, *i.e.*, was already present within the parasite population from Europe, then imported to Brazil. From the molecular point of view, Carnielli et al.^20^ suggested the deletion resulted from homologous recombination, in a genomic region flanked by smaller regions of DNA called repeated sequences (RSs). The circular extrachromosomal element produced was subsequently eliminated by the cell. Once the locus is lost, it remains absent from the population.^20^ Interestingly, despite potentially emerging also among OW strains, the deleted genotype was detected only in American samples, in a higher frequency and widely dispersed compared to the NonDel strains. The higher frequency, wide distribution, combined to restrict circulation to the Americas suggests greater fitness of Del samples to NW conditions. Yet, how a deficient parasite has managed to adapt so successfully in such distinct environment is an open question. The answer might be obtained from the genomic plasticity and peculiar gene expression regulation of *Leishmania*. It is reported that *Leishmania* has a unique, atypical, and unstable genome: it is organised in a peculiar way, *i.e.*, functionally unrelated protein-coding genes are arranged in large polycistronic clusters.^42^ This parasite does have RNA polymerase II promoters, but they are not canonical promoters typically associated with protein-coding genes. The transcription occurs bidirectionally in regions between the polycistronic units, called strand switch regions, and then, the pre-mRNAs formed are processed into mature mRNAs.^42^ ^,^ ^43^ ^,^ ^44^ ^,^ ^45^ This results in weak transcriptional regulation of protein-coding genes.^46^ Moreover, the mosaic aneuploidy,^12^ and the predominantly clonal life cycle with frequent recombination events^47^ increases plasticity. Mosaic aneuploidy is expressed as the variation in the chromosome dosage between cells of the same clonal population, composed of tetrasomic, trisomic, disomic or monosomic cells in variable proportions.^13^ ^,^ ^47^ All *Leishmania* genomes current analysed expressed aneuploidy, but a stable polysomy uniquely in chromosome 31.^48^ ^,^ ^49^ The mosaic aneuploidy leads to changes in gene dosage^16^ and constitutes a strategy for the parasite to adapt to its complex life cycle in the vector and host, as well as to increase drug resistance.^50^ ^,^ ^51^ ^,^ ^52^ ^,^ ^53^ This intrinsic instability in the genome generates phenotypic and genetic variability intra and inter-strains,^12^ ^,^ ^48^ ^,^ ^49^ playing an important role in the biology and evolution of the parasite.^54^

Two distinct levels of biological organisation communicate in evolution: the molecular networks and the genes that encode their organisation and structure.^55^ ^,^ ^56^ Regarding molecular networks, two factors alter protein evolution: (i) translation selection, which refers to the evolutionary selection of characteristics that can increase translation efficiency; and (ii) functional constraint, which refers to the level at which random mutations are removed from the population by natural selection to avoid their deleterious effect on protein function.^57^ The enzyme in a metabolic pathway is also under the protection of functional constraint.^56^ This leads us to speculate that the deletion of 3’NT/NU may alter the purine salvage pathway in *L. infantum*, but does not necessarily represent a risk to the parasite. Despite the loss of 3’-ectonucleotidase activity and the possible reduction of uptake of purines, *Leishmania* can use compensatory means for this function, and/or adapt metabolically to this new nutritional condition. Thus, although the absence of the deleted genes may directly alter phenotype, the associated compensatory mechanisms for gene loss could additionally lead to relevant biological variation. Searching for signals of compensation, Schwabl et al.^11^ found significant copy number variation (CNV) between the NW Del and NonDel strains, in 38 coding regions. Although differences were mostly driven by population structure, after correcting for origin, five coding regions presented significant haplotype somy variance between the Del and NonDel. The annotated loci included nucleoside transporter 1, amastin-like protein and paraflagellar rod protein paralogs.^11^ These findings require further investigation to understand the linkage between the lack of the deleted genes and the CNV report. Amastin-like protein constitute a virulence factor in *Leishmania* and is expressed only in the form of amastigotes;^58^ ^,^ ^59^ nucleoside transporters, in turn, are responsible for mediating transport of nucleosides, nucleobases and therapeutic analogues into the parasite,^60^ ^,^ ^61^ and finally, paraflagellar rod protein are constituents of the paraflagellar rod, a unique network of cytoskeletal filaments that is located next to the axoneme in most trypanosomatids, essential for cell motility.^62^ ^,^ ^63^ Since correlation between CNV and abundance of transcripts has been described, it is expected to observe associated variation in phenotypes.^64^ ^,^ ^65^ To search for CNV and transcripts variation between Del and NonDel may reveal the molecular grounds for increased parasite fitness.

**The sub-chromosomal 12Kb deletion among American *L. infantum* strains and associated features: a genetic marker?**

During a phase 2 clinical trial in Brazil to test efficacy of miltefosine to treat human AVL, the disappointing rate of 60% of clinical cure was reported against the successful 94% rate in India. The reasons behind it remained blurred, until Carnielli et al. revealed the phenotype of natural drug resistance among parasites carrying a 12 Kb deletion in chromosome 31.^20^ The deleted genomic site, MSL was reported as marker for susceptibility to the drug. Patients infected with MSL^-^ parasites presented higher chance to treatment failure. Further on, *in vitro* assays using the same strains confirmed the association between the MSL and the susceptibility to miltefosine. An additional study, however, did not detect any correlation.^66^ The lack of convergence between these studies is a reflex of how controversial genetic markers in *Leishmania* might be. One of the reasons may be, among other molecular traits, the genomic plasticity of these parasites. So, phenotypes such as drug resistance are subjected to the various adaptive strategies the parasite develops to adapt.^48^ ^,^ ^67^ The search for a convergent, unique molecular path is, thus, frequently unproductive. Nevertheless, the deletion is located within the one of more stable chromosome among the mosaic aneuploidies.^21^ The loss of all homologous copies in the locus is, thus, remarkable and might indeed result in stable phenotypic variance. An additional feature to consider regarding the relevance of this natural deletion is the possible compensatory mechanisms developed by the parasite (addressed in next topics), and the annotated function of the four open reading frames within. We believe the main loci at the deleted site that could correlate or lead to vital phenotypic differences between Del an NonDel are the two copies of ecto-3’-nucleotidase/nuclease. The reasons for it are depicted bellow.

The ecto-3’-nucleotidase: why it calls up attention in detriment to the other deleted genes

The ecto-3’-nucleotidase/nuclease (3’NT/NU) enzyme was first described by Gottlieb and Dwyer^68^ through the isolation and purification of surface mem-branes from *L. donovani* promastigotes. Later was found in other trypanosomatids including *L. infantum*.^26^ It is a 43 kDa transmembrane enzyme from the family of class l nucleases, bi-functional, capable of hydrolysing 3’-monophosphorylated nucleotides and nucleic acids, configuring itself as a phosphodiesterase or phosphomonoesterase.^68^ ^,^ ^69^ This enzyme plays an important role in the nutrition of the parasite and in the establishment of infection in the vertebrate host.^22^ *Leishmania* are auxotrophs for purines, unable to synthesise them via *de novo*. For this reason, they make use of the purine salvage pathway to capture preformed nucleotides from the host cell and synthesise their purine nucleotides, necessary for nucleic acid synthesis and other relevant biomolecules.^70^ ^,^ ^71^ 3’NT/NU participates in this process, *i.e.*, the uptake of purines by hydrolysing extracellular nucleotides, conversion into nucleosides such as adenosine,^23^ and transport into the parasite by nucleoside/nucleobase transporters (NT).^61^ In addition to nutritional factors, 3’NT/NU is also associated with the escape of parasites from neutrophil networks (NETs), due to its ability to hydrolyse nucleic acids,^24^ and the ability to infect macrophages.^25^ ^,^ ^26^ Besides this, the fact of 3’NT/NU does not occur in mammals, makes it a potential therapeutic target.^25^ The literature reports that 3’NT/NU has greater activity in infective metacyclic forms and is absent in amastigotes. Due to these characteristics, this enzyme is likely to be more relevant for the parasite in the initial stages of infection and constitutes a virulence factor for *L. infantum*.^22^ ^,^ ^23^ Vieira et al.^26^ have demonstrated that the activity of this enzyme is superior in viscerotropic species of *Leishmania*. *L. infantum* was the species that demonstrated the greatest 3’-nucleotidase activity, followed by *L. donovani*. In the same work, it was also demonstrated that the activity of 3’NT/NU is 10-fold higher than the activity of the enzyme ecto-5’-nucleotidase/nuclease, reinforcing that the deletion of 3’NT/NU cannot be compensated by such path. Lastly, a query in TriTrypDB (Kinetoplastid Informatics Resources, https://tritrypdb.org/tritrypdb/app) reveals that only an extra copy of 3’NT/NU is annotated on chromosome 12, supporting the hypothesis of biological consequence associated to the full deletion of the two copies in chromosome 31. Indeed, the Del strains exhibited reduced/absent 3’NT/NU activity in relation to the NonDel and HTZ strains^11^ indicating that the expression of this enzyme is dependent on the genes deleted. Combined, these facts suggest the existence of relevant differences among Del and NonDel parasites potentially linked to the 3’NT/NU. If such variability is true, distinct hypothesis can be elaborated and should be tested. These will be addressed in the next topics.

***Leishmania*’s 3’NT/NU and the modulation of extracellular adenosine (eADO)**

*Leishmania*’s 3’NT/NU contributes to generate extracellular adenosine (eADO).^26^ This trait ― together with the host’s ectonucleotidases ―, potentially impact purinergic signalling pathway in the vertebrate by the increase of extracellular level of purines.^54^ ^,^ ^72^ The host’s ectonucleotidases CD39 and CD73 are highly expressed in situations of injury, stress and infection.^73^ When the cell is under one of these conditions, ATP can be released into the extracellular environment as a signal of damage, alerting the host’s immune system.^74^ This extracellular ATP activates receptors of the P1-type purinergic signalling pathway,^72^ triggering an inflammatory response characterised by the activation of macrophages, dendritic cells, and the secretion of pro-inflammatory cytokines.^75^ In this way, CD39 and CD73 are expressed in cell membranes to regulate extracellular levels of ATP, hydrolysing it into adenosine (ADO).^54^ Certain *Leishmania* species, including *L. infantum*, can subvert the host’s inflammatory response by inducing the cell to produce and release more ATP into the extracellular environment, positively regulating the expression of CD39 and CD73 and generating sustained levels of ADO.^76^ ^,^ ^77^ ^,^ ^78^ Extracellular ADO will activate two G protein-coupled adenosine receptors, A2AR (high affinity) and A2BR (low affinity), culminating in the inhibition of the secretion of pro-inflammatory cytokines, inhibition of nitric oxide (NO) production and the microbicidal effects of macrophages, generating an anti-inflammatory milieu conducive to parasite survival.^77^ ^,^ ^79^ ^,^ ^80^ The activation of A2AR receptors also negatively regulates the migration and activation of neutrophils that are induced by Th1 cells, impairing their influx. The absence of A2AR receptor leads to a strong Th1 response and a decrease in spleen and liver tissue parasitism, suggesting that this receptor and its respective activation are important for the establishment of visceral infection by *Leishmania*.^76^ This gives us a glimpse of the importance of eADO generation and its role in the success of infection by this parasite. Thus, two main points must be highlighted in this regard: 1) ADO is a signalling molecule that directly interferes with the host’s immune response and influences the survival and visceralisation of *Leishmania infantum*; 2) *Leishmania* contributes to modulate the extracellular levels of ADO through the hydrolysis of 3’-AMP by its 3’-NT/NU enzymes;^22^ ^,^ ^26^ 3) Ultimately, Del and NonDel *L. infantum* strains exhibiting distinct 3’-NT/NU activity^11^ and thus, variable abilities to generate eADO might consequently express distinct virulence.

The biological effect of eADO on *Leishmania* is not restricted to the vertebrate host. Serafim et al.^81^ demonstrate that ADO restriction triggers *Leishmania* differentiation into infective metacyclic forms within the sandfly and *in vitro*. Cultures of promastigotes treated with an antagonist of mammalian adenosine receptors, CGS 15943 (also capable of inhibiting adenosine uptake in *Leishmania*), presented higher metacyclogenesis. When CGS-treated promastigotes were cultured in medium poor in nutrients, the parasites were not able to proliferate, and metacyclogenesis was triggered; the events were reversed by the addition of adenosine. They also confirmed in experiments with *Lu. longipalpis*, that infected insects fed with ADO and sucrose presented reduced number of metacyclic. These findings confirmed that ADO is an essential nutrient for the multiplication of parasites, and its absence triggers *Leishmania* differentiation even in the in-vertebrate host. Moreover, it is reported that, *Lu. longipalpis* and *Phlebotomus* (vectors in the OW) differ, among other aspect, in the constituents of their saliva. ADO and AMP were present in the salivary extract of *Phlebotomus papatasi* e *Phlebotomus duboscqi*, but, curiously, not in *Lu. longipalpis*.^82^ Exporting the reports from literature to the current scenario of Del and NonDel circulation, we raise the question whether the fitness of these strains differ in intra vectorial stage and thus affect the frequency and distribution of these genotypes in Brazil.

3’NT/NU association to miltefosine susceptibility

The association described between infection by Del parasites and patients presenting miltefosine treatment failure^20^ was further explored by the same authors using CRISPR Cas9.^83^ The connection between phenotype / genotype was validated by the addback parasites and, importantly, authors identified the genes within the deleted site (MSL) responsible to the miltefosine susceptibility. Knockout cell lines were generated by CRISPR Cas9, for each of the genes deleted in MSL: ∆nuc1 (NUC1 - deletion of the 3’-nucleotidase/nuclease), ∆nuc2 (NUC2 - deletion of the 3’-nucleotidase/nuclease precursor), ∆hlp (HLP - deletion of the helicase-like protein) and ∆tei (TEI - deletion of 3,2-trans-enoyl-CoA isomerase), for both deleted 3’-nucleotidase/nuclease genes, ∆nuc1/nuc2, and for deletion of the entire MSL locus, ∆msl. Using these strains, the authors evaluated different parameters related to the resistance of Del strains (MSL^-^) to miltefosine previously described^20^ and concluded that isogenic cell lines with the complete deletion ∆msl or with the 3’NT/NU genes deleted ∆nuc1/ nuc2, showed a significant reduction in sensibility to miltefosine, in both amastigote and promastigote forms. It was also observed that these same mutants showed a better ability to control the disturbances caused by miltefosine in the lipidome, reinforcing that 3’NT/NU is a component crucial among the deleted genes for the manifestation of the miltefosine-susceptible phenotype.

Remarks and open questions

The study of the deletion-carrying strains described in Carnielli et al.^20^ and Schwabl et al.,^11^ represent a unique opportunity to advance the understanding of adaptive and evolutionary molecular strategies in *Leishmania*. The biological effects and compensatory responses at genomic, post-transcriptional and translational levels likely contributed to fitness gain for the parasite, which in turn may lead to key variation of epidemiological outcomes. As an example, is the association between Del parasites, (more precisely, lacking 3’NU/NT) and the reduced susceptibility to miltefosine. The drug, although not used in Brazil to treat visceral leishmaniasis in human, was recently approved to treat infected dogs. As the main urban reservoir, a treated dog infected with the not-susceptible Del genotype would continue to harbour significant parasite load, and thus remain a source of infection for the vector. Another major point to address is why Del parasites are widely dispersed and more frequent. Both vertebrate and invertebrate hosts must be considered as major players in this scenario. For vertebrate host, it is plausible to expect reduced virulence during infection by Del strains due to the nature of the deleted genes, especially 3’NU/NT, associated with parasite viability and infectivity. The reduced CNV for Amastin in Del samples (genes also relevant for infection), additionally supports the idea of altered infectivity in these strains. A reasonable hypothesis, thus, is that dogs infected by Del *L. infantum* progress as asymptomatic, remaining undetected by surveillance services, ultimately contributing to keep the circulation, and spread of these parasites. For the invertebrate, it is important to cogitate coevolutionary interactions between parasite and phlebotomine genotypes that may have shaped variable transmission cycles. Differences in saliva content, for instance, which includes presence of ADO, AMP and ectonucleotidases among vector species, may act as important factors for the transmissibility of *L. infantum* Del and NonDel. By addressing the presented open questions associated to the cocirculation of the distinct genotypes of *L. infantum* in Brazil, researchers will generate valuable data on adaptive molecular mechanisms and drug susceptibility of *Leishmania*, the parasite-hosts interaction, and, ultimately, transmission control.

## References

[1] WHO (2023). Leishmaniasis. https://www.who.int/health-topics/leishmaniasis#tab=tab_1.

[2] WHO (2023). Number of cases of cutaneous leishmaniasis reported Data by country. https://apps.who.int/gho/data/node.main.NTDLEISHCNUM?lang=en.

[3] WHO (2023). Number of cases of visceral leishmaniasis reported data by country. https://apps.who.int/gho/data/node.main.NTDLEISHVNUM?lang=en.

[4] Akhoundi M, Kuhls K, Cannet A, Votýpka J.Marty P.Delaunay P (2016). A historical overview of the classification, evolution, and dispersion of Leishmania parasites and sandflies. PLoS Negl Trop Dis.

[5] Steverding D (2017). The history of leishmaniasis. Parasit Vectors.

[6] Altamirano-Enciso AJ, Marquez MG, Ferreira MC, Rocha-Santos AA, Borja-Flores MB (2003). Sobre a origem e dispersão das leishmanioses cutânea e mucosa com base em fontes históricas pré e pós-colombianas. Hist Cienc Saude-Manguinhos.

[7] Momen H, Cupolillo E (2000). Speculations on the origin and evolution of the genus Leishmania. Mem Inst Oswaldo Cruz.

[8] Momen H, Cupolillo E, Grimaldi JG (1993). Molecular evidence for the importation of Old World Leishmania into the Americas. Biol Res.

[9] Leblois R, Kuhls K, François O, Schönian G, Wirth T (2011). Guns, germs and dogs on the origin of Leishmania chagasi. Infect Genet Evol.

[10] Boité MC, Späth GF, Bussotti G, Porrozzi R, Morgado FN, Llewellyn M (2020). Trans-atlantic spillover deconstructing the ecological adaptation of Leishmania infantum in the Americas. Genes.

[11] Schwabl P, Boité MC, Bussotti G, Jacobs A, Andersson B, Moreira O (2021). Colonization and genetic diversification processes of Leishmania infantum in the Americas. Commun Biol.

[12] Sterkers Y, Lachaud L, Bourgeois N, Crobu L, Bastien P, Pagès M (2012). Novel insights into genome plasticity in eukaryotes Mosaic aneuploidy in Leishmania. Mol Microbiol.

[13] Bussotti G, Li B, Pescher P, Vojtkova B, Louradour I, Pruzinova K (2023). Leishmania allelic selection during experimental sand fly infection correlates with mutational signatures of oxidative DNA damage. Proc Natl Acad Sci USA.

[14] Akopyants NS, Kimblin N, Secundino N, Patrick R, Peters N, Lawyer P (2009). Demonstration of genetic exchange during cyclical development of Leishmania in the sand fly vector. Science.

[15] Rogers MB, Downing T, Smith BA, Imamura H, Sanders M, Svobodova M (2014). Genomic confirmation of hybridization and recent inbreeding in a vector-isolated Leishmania population. PLoS Genet.

[16] Lachaud L, Bourgeois N, Kuk N, Morelle C, Crobu L, Merlin G (2014). Constitutive mosaic aneuploidy is a unique genetic feature widespread in the Leishmania genus. Microbes Infect.

[17] Franssen SU, Durrant C, Stark O, Moser B, Downing T, Imamura H (2020). Global genome diversity of the Leishmania donovani complex. Elife.

[18] Imamura H, Downing T, Van den Broeck F.Sanders MJ.Rijal S.Sundar S (2016). Evolutionary genomics of epidemic visceral leishmaniasis in the Indian subcontinent. Elife.

[19] Guerbouj S, Guizani I, Speybroeck N, Le Ray D, Dujardin JC (2001). Genomic polymorphism of Leishmania infantum A relationship with clinical pleomorphism?. Infect Genet Evol.

[20] Carnielli JBT, Crouch K, Forrester S, Silva VC, Carvalho SFG, Damasceno JD (2018). A Leishmania infantum genetic marker associated with miltefosine treatment failure for visceral leishmaniasis. EBioMedicine.

[21] Negreira GH, Monsieurs P, Imamura H, Maes I, Kuk N, Yagoubat A (2022). High throughput single-cell genome sequencing gives insights into the generation and evolution of mosaic aneuploidy in Leishmania donovani. Nucleic Acids Res.

[22] Freitas-Mesquita AL, Meyer-Fernandes JR (2017). 3&apos;Nucleotidase/Nuclease in protozoan parasites molecular and biochemical properties and physiological roles. Exp Parasitol.

[23] Paletta-Silva R, Vieira DP, Vieira-Bernardo R, Majerowicz D, Gondim KC, Vannier-Santos MA (2011). Leishmania amazonensis characterization of an ecto-3'-nucleotidase activity and its possible role in virulence. Exp Parasitol.

[24] Guimarães-Costa AB, DeSouza-Vieira TS, Paletta-Silva R, Freitas-Mesquita AL, Meyer-Fernandes JR, Saraiva EM (2014). 3&apos;-Nucleotidase/Nuclease activity allows Leishmania parasites to escape killing by neutrophil extracellular traps. Infect Immun.

[25] Freitas-Mesquita AL, Gomes MT, Vieira DP, Paes-Vieira L, Nascimento MTC, Lopes AHCS (2016). Inhibitory effects promoted by 5&apos;-nucleotides on the ecto-3&apos;-nucleotidase activity of Leishmania amazonensis. Exp Parasitol.

[26] Vieira DP, Paletta-Silva R, Saraiva EM, Lopes AHCS, Meyer-Fernandes JR (2011). Leishmania chagasi an ecto-3&apos;-nucleotidase activity modulated by inorganic phosphate and its possible involvement in parasite-macrophage interaction. Exp Parasitol.

[27] Dantas-Torres F (2007). The role of dogs as reservoirs of Leishmania parasites, with emphasis on Leishmania (Leishmania) infantum and Leishmania (Viannia) braziliensis. Vet Parasitol.

[28] Quinnell RJ, Courtenay O (2009). Transmission, reservoir hosts and control of zoonotic visceral leishmaniasis. Parasitology.

[29] Cecílio P, Cordeiro-da-Silva A, Oliveira F (2022). Sand flies basic information on the vectors of leishmaniasis and their interactions with Leishmania parasites. Commun Biol.

[30] Tarallo VD, Dantas-Torres F, Lia RP, Otranto D (2010). Phlebotomine sand fly population dynamics in a leishmaniasis endemic peri-urban area in southern Italy. Acta Trop.

[31] Carvalho De Resende M.Camargo MC.Reis J.Vieira M.Celi R.Filho SD (2006). Seasonal variation of Lutzomyia longipalpis in Belo Horizonte, State of Minas Gerais. Rev Soc Bras Med Trop.

[32] Missawa NA, Maciel GB, Michalsky ÉM, Dias ES (2011). Evidence of transmission of visceral leishmaniasis by Lutzomyia cruzi in the municipality of Jaciara, State of Mato Grosso, Brazil. Rev Soc Bras Med Trop.

[33] Alexandre J, Sadlova J, Lestinova T, Vojtkova B, Jancarova M, Podesvova L (2020). Experimental infections and co-infections with Leishmania braziliensis and Leishmania infantum in two sand fly species, Lutzomyia migonei and Lutzomyia longipalpis. Sci Rep.

[34] De Souza NA, Brazil RP, Araki AS (2017). The current status of the Lutzomyia longipalpis (Diptera Psychodidae: Phlebotominae) species complex. Mem Inst Oswaldo Cruz.

[35] Maingon RDC, Ward RD, Hamilton JGC, Bauzer LGSR, Peixoto AA (2008). The Lutzomyia longipalpis species complex does population sub-structure matter to Leishmania transmission?. Trends Parasitol.

[36] Ready PD (2013). Biology of phlebotomine sand flies as vectors of disease agents. Annu Rev Entomol.

[37] Ferreira GEM, dos Santos BN, Dorval MEC, Ramos TPB, Porrozzi R, Peixoto AA (2012). The genetic structure of Leishmania infantum populations in Brazil and its possible association with the transmission cycle of visceral leishmaniasis. PLoS One.

[38] Andrade-Filho JD, Scholte RGC, Amaral ALG, Shimabukuro PHF, Carvalho OS, Caldeira RL (2017). Occurrence and probability maps of Lutzomyia longipalpis and Lutzomyia cruzi (Diptera Psychodidae: Phlebotominae) in Brazil. J Med Entomol.

[39] Oliveira S, Ribeiro A, Pade P, Hoffman V (1998). Incrimination of Lutzomyia cruzi as a vector of American visceral leishmaniasis. Med Vet Entomol.

[40] Santos MFC, Ribolla PEM, Alonso DP, Andrade-Filho JD, Casaril AE, Ferreira AMT (2013). Genetic structure of Lutzomyia longipalpis populations in Mato Grosso do Sul, Brazil, based on microsatellite markers. PLoS One.

[41] Romano A, Inbar E, Debrabant A, Charmoy M, Lawyer P, Ribeiro-Gomes F (2014). Cross-species genetic exchange between visceral and cutaneous strains of Leishmania in the sand fly vector. Proc Natl Acad Sci USA.

[42] Grünebast J, Clos J (2020). Leishmania responding to environmental signals and challenges without regulated transcription. Comput Struct Biotechnol J.

[43] Morel CM, Acharya T, Broun D, Dangi A, Elias C, Ganguly NK (2005). Health innovation networks to help developing countries address neglected diseases. Science.

[44] Martínez-Calvillo S, Nguyen D, Stuart K, Myler PJ (2004). Transcription initiation and termination on Leishmania major chromosome 3. Eukaryot Cell.

[45] Martínez-Calvillo S, Nguyen D, Stuart K, Myler PJ (2004). Transcription of Leishmania major Friedlin chromosome 1 initiates in both directions within a single region. Mol Cell.

[46] Clayton CE (2002). Life without transcriptional control from fly to man and back again. EMBO J.

[47] Tibayrenc M, Ayala FJ (2021). Leishmania and the model of predominant clonal evolution. Microorganisms.

[48] Ubeda JM, Légaré D, Raymond F, Ouameur AA, Boisvert S, Rigault P (2008). Modulation of gene expression in drug resistant Leishmania is associated with gene amplification, gene deletion and chromosome aneuploidy. Genome Biol.

[49] Sterkers Y, Crobu L, Lachaud L, Pagès M, Bastien P (2014). Parasexuality and mosaic aneuploidy in Leishmania alternative genetics. Trends Parasitol.

[50] Rogers MB, Hilley JD, Dickens NJ, Wilkes J, Bates PA, Depledge DP (2011). Chromosome and gene copy number variation allow major structural change between species and strains of Leishmania. Genome Res.

[51] Sterkers Y, Lachaud L, Crobu L, Bastien P, Pagès M (2011). FISH analysis reveals aneuploidy and continual generation of chromosomal mosaicism in Leishmania major. Cell Microbiol.

[52] Dumetz F, Imamura H, Sanders M, Seblova V, Myskova J, Pescher P (2017). Modulation of aneuploidy in. Leishmania donovani during adaptation to different in vitro and in vivo environments and its impact on gene expression. mBio.

[53] Papadopoulou B, Ouellette M, Laffitte MCN, Leprohon P (2016). Plasticity of the Leishmania genome leading to gene copy number variations and drug resistance. F1000Res.

[54] Robson SC, Sévigny J, Zimmermann H (2006). The E-NTPDase family of ectonucleotidases Structure function relationships and pathophysiological significance. Purinergic Signal.

[55] Subramanian A, Sarkar RR (2018). Evolutionary perspectives of genotype-phenotype factors in Leishmania metabolism. J Mol Evol.

[56] Vitkup D, Kharchenko P, Wagner A (2006). Influence of metabolic network structure and function on enzyme evolution. Genome Biol.

[57] Zhang J, Yang JR (2015). Determinants of the rate of protein sequence evolution. Nat Rev Genet.

[58] Wu Y, El Fakhry Y, Sereno D, Tamar S, Papadopoulou B (2000). A new developmentally regulated gene family in Leishmania amastigotes encoding a homolog of amastin surface proteins. Mol Biochem Parasitol.

[59] Rochette A, McNicoll F, Girard J, Breton M, Leblanc É, Bergeron MG (2005). Characterization and developmental gene regulation of a large gene family encoding amastin surface proteins in Leishmania spp. Mol Biochem Parasitol.

[60] Boswell-Casteel RC, Hays FA (2017). Equilibrative nucleoside transporters - A review. Nucleosides Nucleotides Nucleic Acids.

[61] Stein A, Vaseduvan G, Carter NS, Ullman B, Landfear SM, Kavanaugh MP (2003). Equilibrative nucleoside transporter family members from Leishmania donovani are electrogenic proton symporters. J Biol Chem.

[62] Saravia NG, Hazbón MH, Osorio Y, Valderrama L, Walker J, Santrich C (2005). Protective immunogenicity of the paraflagellar rod protein 2 of Leishmania mexicana. Vaccine.

[63] Maga JA, Sherwin T, Francis S, Gull K, LeBowitz JH (1999). Genetic dissection of the Leishmania paraflagellar rod, a unique flagellar structure. J Cell Sci.

[64] Prieto Barja P, Pescher P, Bussotti G, Dumetz F, Imamura H, Kedra D (2017). Haplotype selection as an adaptive mechanism in the protozoan pathogen Leishmania donovani. Nat Ecol Evol.

[65] Iantorno SA, Durrant C, Khan A, Sanders MJ, Beverley SM, Warren WC (2017). Gene expression in. Leishmania is regulated predominantly by gene dosage. mBio.

[66] Espada CR, Levatti EVC, Boité MC, Lamounier D, Alvar J, Cupolillo E (2021). In vitro susceptibility to miltefosine of Leishmania infantum (Syn L. chagasi) isolates from different geographical areas in Brazil. Microorganisms.

[67] Downing T, Imamura H, Decuypere S, Clark TG, Coombs GH, Cotton JA (2011). Whole genome sequencing of multiple Leishmania donovani clinical isolates provides insights into population structure and mechanisms of drug resistance. Genome Res.

[68] Gottlieb M, Dwyer DM (1983). Evidence for distinct 5'- and 3'-nucleotidase activities in the surface membrane fraction of Leishmania donovani promastigotes. Mol Biochem Parasitol.

[69] Dwyer DM, Gottlieb M (1984). Surface membrane localization of 3'- and 5'-nucleotidase activities in Leishmania donovani promastigotes. Mol Biochem Parasitol.

[70] Marr JJ, Berens RL, Nelson DJ (1978). Purine metabolism in Leishmania donovani and Leishmania braziliensis. Biochim Biophys Acta.

[71] Gottlieb M (1989). The surface membrane 3'-nucleotidase/nuclease of trypanosomatid protozoa. Parasitol Today.

[72] Abbracchio MP, Burnstock G (1994). Purinoceptors are there families of P2X and P2Y purinoceptors?. Pharmacol Ther.

[73] Haskó G, Linden J, Cronstein B, Pacher P (2008). Adenosine receptors therapeutic aspects for inflammatory and immune diseases. Nat Rev Drug Discov.

[74] Di Virgilio F (2005). Purinergic mechanism in the immune system a signal of danger for dendritic cells. Purinergic Signal.

[75] Junger WG (2011). Immune cell regulation by autocrine purinergic signalling. Nat Rev Immunol.

[76] Lima MHF, Sacramento LA, Quirino GFS, Ferreira MD, Benevides L, Santana AKM (2017). Leishmania infantum parasites subvert the host inflammatory response through the adenosine A2A receptor to promote the establishment of infection. Front Immunol.

[77] Basu M, Gupta P, Dutta A, Jana K, Ukil A (2020). Increased host ATP efflux and its conversion to extracellular adenosine is crucial for establishing Leishmania infection. J Cell Sci.

[78] Zimmermann H (2000). Extracellular metabolism of ATP and other nucleotides. Naunyn Schmiedebergs Arch Pharmacol.

[79] Figueiredo AB, Serafim TD, Marques-da-Silva EA, Meyer-Fernandes JR, Afonso LCC (2012). Leishmania amazonensis impairs DC function by inhibiting CD40 expression via A2B adenosine receptor activation. Eur J Immunol.

[80] Fredholm BB, Arslan G, Halldner L, Kull B, Schulte G, Wasserman W (2000). Structure and function of adenosine receptors and their genes. Naunyn Schmiedebergs Arch Pharmacol.

[81] Serafim TD, Figueiredo AB, Costa PAC, Marques-da-Silva EA, Gonçalves R, de Moura SAL (2012). Leishmania metacyclogenesis is promoted in the absence of purines. PLoS Negl Trop Dis.

[82] Gomes R, Oliveira F (2012). The immune response to sand fly salivary proteins and its influence on Leishmania immunity. Front Immunol.

[83] Carnielli JBT, Dave A, Romano A, Forrester S, de Faria PR, Monti-Rocha R (2022). 3&apos;Nucleotidase/nuclease is required for Leishmania infantum clinical isolate susceptibility to miltefosine. EBioMedicine.

